# Examining Psychedelic-Induced Changes in Social Functioning and Connectedness in a Naturalistic Online Sample Using the Five-Factor Model of Personality

**DOI:** 10.3389/fpsyg.2021.749788

**Published:** 2021-11-25

**Authors:** Brandon Weiss, Victoria Nygart, Lis Marie Pommerencke, Robin L. Carhart-Harris, David Erritzoe

**Affiliations:** ^1^Psychedelic Research Group, Division of Psychiatry, Department of Brain Sciences Department of Medicine, Imperial College London, London, United Kingdom; ^2^National Institute of Public Health, University of Southern Denmark, Odense, Denmark; ^3^Psychedelics Division, Neuroscape, Department of Neurology, University of California, San Francisco, San Francisco, CA, United States

**Keywords:** psychedelic, personality change, prospective, social functioning, Five-Factor Model

## Abstract

The present study examines prospective changes in personality traits relevant to social functioning as well as perceived social connectedness in relation to the naturalistic use of psychedelic compounds in an online volunteer sample. The study also examined the degree to which demographic characteristics, social setting, baseline personality, and acute subjective factors (e.g., emotional breakthrough experiences) influenced trajectories of personality and perceived social connectedness. Participants recruited online completed self-report measures of personality and social connectedness at three timepoints (baseline, 2weeks post-experience, 4weeks post-experience). Linear mixed models were used to examine changes in outcomes and the moderation of these outcomes by covariates. The most substantive changes were reductions in the personality domains Neuroticism, and increases in Agreeableness and social connectedness. Notably, reductions in Neuroticism and increases in Agreeableness covaried over time, which may be suggestive of common processes involving emotion regulation. Preliminary evidence was found for a specific effect on a component of Agreeableness involving a critical and quarrelsome interpersonal style. Although moderation by demographic characteristics, social setting, baseline personality, and acute factors generally found limited support, baseline standing on Neuroticism, perspective taking, and social connectedness showed tentative signs of amplifying adaptive effects on each trait, respectively. Our findings hold implications for the potential use of psychedelics for treating interpersonal elements of personality pathology as well as loneliness.

## Introduction

Research on serotonergic psychedelics has accelerated in the last decade (Carhart-Harris and Goodwin, 2017) due to promising demonstrations of psychotherapeutic effects, and relaxed legal restriction on scientific investigation (Nutt et al., 2013). Research in this area has mainly focused on contributions to individual utility, including gains in personal well-being (e.g., Griffiths et al., 2011), personality (e.g., Erritzoe et al., 2018), and remediation of mental health disorders including alcohol misuse (Bogenschutz et al., 2015), major and resistant depression (e.g., Carhart-Harris et al., 2016a, 2021; Davis et al., 2020), and end of life anxiety/depression (e.g., Griffiths et al., 2016). A smaller and growing area of psychedelic research has explored how psychedelic compounds may confer value to both individuals and collectives through such outcomes as enhanced cooperation, trust, and social connectedness within interpersonal systems (referred to here as collective functioning).

Collective functioning can conceivably be enhanced directly through mutual participation in group-format psychedelic experiences (e.g., ayahuasca ceremony, group recreation; e.g., Kettner et al., 2021), and/or indirectly *via* the development of traits in individuals related to social functioning. The present study focuses on the latter path, wherein the link between psychedelic use and collective functioning may be mediated by enhanced social functioning at the individual level. Social functioning is defined as the ability to fulfill important roles within environments of social activity, work, and relationships (Bosc, 2000), but will be used more broadly here to describe personality traits that subserve harmonious relationships including empathic ability, prosociality, low anger reactivity, and moral reasoning. Improved social functioning is assumed to promote positive individual and collective outcomes through augmenting interpersonal trust, social connectedness, relationship longevity, and emotional fulfillment in social interaction. The purpose of this study is to investigate the degree to which naturalistic use of psychedelic compounds results in adaptive changes in personality traits relevant to social functioning and perceived social connectedness. Because personality change effects may depend on other measurable factors, we also examine the degree to which predisposing factors, such as demographic characteristics and baseline personality, and acute subjective factors, such as non-ordinary states of affect and consciousness during psychedelic experience, moderate changes.

A useful framework for our examination lies in the Five-Factor Model (FFM; Costa and McCrae, 1992) of personality, which includes three personality domains that possess empirical support for subserving social functioning. Neuroticism describes a disposition toward negative emotionality and stress reactivity; Extraversion describes a disposition toward positive emotionality, reward sensitivity, and receiving social attention; and Agreeableness describes a prosocial and communal (versus antagonistic) tendency to be considerate of others’ needs, desires, and feelings, and is neurocognitively associated with social information processing (e.g., DeYoung, 2010; DeYoung et al., 2010). In the context of relationships, low Extraversion (being withdrawn) and low Agreeableness are associated with peer rejection (Newcomb et al., 1993), and higher levels of Extraversion are associated with social acceptance and network size, popularity, and dating variety (e.g., Anderson et al., 2001; Paunonen, 2003; Feiler and Kleinbaum, 2015). In the context of romantic relationships, Agreeableness has shown relations with relationship satisfaction (e.g., Cramer and Jowett, 2010; Kimmes et al., 2014). Consistent with this, Neuroticism and low Agreeableness have shown predictive relations with relationship dissatisfaction, conflict, and dissolution (e.g., Karney and Bradbury, 1995; Donnellan et al., 2004; Weiss et al., 2018), outcomes thought to be mediated by a positive feedback loop involving negative emotion and relationship distress (Robins et al., 2002).

In the context of larger groups, Agreeableness and Extraversion has been found to be related to cooperation, and Agreeableness shows evidence of being inversely related to hypercompetition (Ross et al., 2003). Agreeableness may be most relevant to social and collective functioning as it involves higher functioning in empathy, a disposition toward prosociality (or antisociality at its opposite pole), and is associated with relationship stability. Agreeableness bears substantial overlap with affective and cognitive empathy, which, respectively, describe capacities to infer the emotional experience of others and to occupy the mental perspectives of others (e.g., Melchers et al., 2016). Agreeableness was also found to be elevated among young adult moral exemplars (Matsuba and Walker, 2004). At its opposite pole, low Agreeableness (or Antagonism) is thought to reflect the core of personality disorders such as Narcissistic Personality Disorder, Antisocial Personality Disorder, and psychopathy (e.g., Widiger et al., 2012), as well as Antagonistic Externalizing, one of two Externalizing spectra within the Hierarchical Taxonomy of Psychopathology (Kotov et al., 2017), a data-driven and integrative model of psychopathology. Antagonistic Externalizing describes tendencies toward engaging in criminal and antisocial acts involving theft, destruction of property, physical aggression toward others, and violations of rules of conduct (Widiger et al., 2012; Kotov et al., 2017). In sum, Neuroticism, Extraversion, and Agreeableness may be particularly relevant targets of inquiry when examining the effects of psychedelic compounds on social functioning.

There are four sources of empirical evidence that best inform the presence of a link between psychedelic use and enhanced social functioning. These include literature examining the effects of psychedelic compounds on FFM personality, social cognition, social connectedness, and moral behavior. The most relevant evidence to the present study is located in the personality literature. Eleven studies have prospectively examined long-term psychedelic-induced change in personality (e.g., MacLean et al., 2011; Madsen et al., 2020). Of these studies, five have shown evidence of adaptive change in Neuroticism or convergent constructs [e.g., Temperament and Character Inventory Harm Avoidance (characterized by worry)] (e.g., Barbosa et al., 2009; Erritzoe et al., 2018). Two studies have shown evidence of self-reported change in Extraversion (Erritzoe et al., 2018; Weiss et al., 2021), with change notably reflected in facets relevant to social functioning (Warmth/Friendliness, Gregariousness). Lastly, three studies have shown qualified evidence of increased Agreeableness (Carhart-Harris et al., 2016b; Netzband et al., 2020; Weiss et al., 2021). Although peripherally relevant to our focus on social functioning, seven studies have found evidence for change in Openness (e.g., MacLean et al., 2011; Rocha et al., 2021; Weiss et al., 2021), whereas only one has found evidence of change in Conscientiousness (Barrett et al., 2020). Of note, some studies have found no meaningful evidence of longer-term change in personality (e.g., Schmid and Liechti, 2018; Rocha et al., 2021). In addition, numerous clinical studies, including randomized placebo-controlled trials, have provided support for an antidepressant effect (Andersen et al., 2021). Given that many psychopathological symptoms are increasingly conceptualized as maladaptive variants of basic personality dimensions and share the same structure (Widiger et al., 2012; Kotov et al., 2017), these latter results may be relevant to change in FFM Neuroticism and Extraversion. In sum, long-term psychedelic-induced changes in personality domains relevant to social functioning have been observed, but findings remain somewhat inconsistent. This inconsistency is likely to stem from multiple sources including study variability in sample size, sample type (general population, clinical, healthy), length of follow-up, conditions of administration, and inner experiences during the acute effects of the compounds. One major limitation of the existing literature is that sample size for prospective studies has been low (N=25), making it difficult to arrive at precise estimates of change (given high standard error), or to examine moderating factors predictive of change (given insufficient statistical power).

The second source of evidence lies in the area of social cognition. Existing findings are suggestive that psychedelic compounds modulate social cognition for up to a week following use. Specifically, behavioral and neurophysiological findings have shown that 5-HT_2A_ receptor agonists acutely produce attenuated processing of social rejection (based on self-report and dorsal anterior cingulate cortex; Preller et al., 2016) and negative facial expressions (based on self-report and N170, P300 evoked-response-potential; Kometer et al., 2012; Dolder et al., 2016), which may be suggestive of downstream emotional resilience in social settings, greater levels of social approach, and lower avoidance. Studies have also been supportive of acute increases in emotional empathy (Dolder et al., 2016; Holze et al., 2021a). More recent research has shown evidence of post-acute changes in amygdala blood oxygen-level dependent response to emotional face stimuli, though the directionality of effects has differed across studies. One study showed reduced amygdala activations lasting at least a week following acute effects, but not longer than 1month (Barrett et al., 2020). Another, examining patients with treatment-resistant depression, showed increased amygdala activations one day post dosing with psilocybin (Roseman et al., 2018). Collectively, these findings suggest that psychedelic compounds alter social cognition-related neural functioning sub- and post-acutely.

A third source of evidence observes changes in perceived social connectedness during and following psychedelic experience, a phenomenon that may be linked to changes in social cognition just described. In a large cross-sectional examination of festival-goers, Forstmann et al. (2020) showed that psychedelic compounds were unique among substances in producing increased perceptions of social connectedness with other human beings broadly and with fellow event attendees. However, these effects obtained only for participants who endorsed using psychedelic compounds within the preceding 24h, but not earlier, suggesting that social connectedness effects may be temporary. Although long-term changes in self-reported interpersonal closeness were found 6months following the acute effects of psilocybin in one study (Griffiths et al., 2018), these findings remain tentative in view of failure to find a significant difference from the control group. Findings of increased perceived social connectedness are consistent with at least two sources of qualitative data demonstrating an enhanced sense of connectedness to significant others and other human beings, marked by openness to others, sociability, and authentic expression (Belser et al., 2017; Watts et al., 2017). Changes in perceived social connectedness may also be one manifestation of a general sense of connectedness that is described in qualitative reports (Belser et al., 2017) and instantiated in measures of mystical-type experience [i.e., unitive consciousness (or a sense of “oneness” or “unity”)].

A fourth source of evidence involves a small body of literature investigating whether psychedelic compounds promote moral reasoning and prosocial behavior. Findings from this body of literature have however been strongly mixed. Among the supportive findings, there is evidence of a long-term self-reported increase in altruism and positive social effects from three small prospective studies (Dolder et al., 2016; Griffiths et al., 2018; Schmid and Liechti, 2018), the latter of which demonstrated changes over and above a control group. Furthermore, in two large observational samples of criminal offenders, a diagnosis of hallucinogen use disorder was predictive of lower recidivism, violations of supervisory rules in a correctional setting, and future arrests involving intimate partner violence (Hendricks et al., 2014; Walsh et al., 2016). These findings may be suggestive of long-term reductions in Antagonistic Externalizing and Antagonism following psychedelic use. Nevertheless, despite being prospective studies, it is conceivable that other explanations can account for these observations, most notably that participants inclined to use psychedelic compounds already differed from other participants on traits associated with these outcomes before using. These findings stand in contrast to others that fail to support a link between psychedelic use and moral reasoning or behavior.

Although the 5-HT receptor system shows evidence of an involvement in harm aversion (self & other) and deontological (versus utilitarian) moral reasoning (Crockett et al., 2010, 2015), 5-HT_2A_ receptor agonists were not associated with greater aversion to harm others in the only existing examination containing a social moral dilemma task (though sample size was low, N=33; Pokorny et al., 2017). Furthermore, when offered the opportunity to express prosocial behavior through the allocation of redeemable lottery tickets and money to strangers, psychedelic users (within the aforementioned festival context) were no more likely to do so than other participants (Forstmann et al., 2020). Psychedelic users in this context also did not differ from others in ascribing moral praiseworthiness to deeds on the basis of the value of the deed’s outcome for others (versus the hedonic value of the deed for the agent). These findings are also consistent with null observer-rated changes in interpersonal perceptiveness, caring, anger expression, and compassion/social concern in a small prospective study (Griffiths et al., 2018). In sum, evidence is mixed with respect to psychedelic-induced improvements in moral reasoning and prosocial behavior. It may also be possible that substantive change occurs selectively in individuals higher in Antagonism (i.e., low Agreeableness).

The present study builds upon these findings through examining change in FFM personality domains relevant to social functioning as well as perceptions of social connectedness in a sample of 148 individuals recruited online. Our naturalistic approach enabled the collection of a larger sample through greater cost-effectiveness relative to laboratory-based studies, and provides an opportunity to gain insight into the effects of psychedelic use in the general population. Understanding how psychedelic experiences unfold outside of a controlled laboratory or shamanic setting—where harm reduction practices may be lacking—is ethically important as decriminalization/legalization policies expand use above and beyond already high prevalence in the West (Krebs and Johansen, 2013a,b; Winstock, 2017). In our study, a web-based survey—embedded within a purpose-built website—was used to measure outcomes and relevant moderators across five timepoints—including (1) 1week before, (2) within 1day before, (3) within a few days after, (4) 2weeks after, and (5) 4weeks after a psychedelic experience—among which study outcomes were measured 1week before, 2weeks after, and 4weeks after. Using an integrated FFM framework, we also included outcomes conceptually and empirically related to FFM domains (e.g., measures of compassion and affective empathy enrich measurement of FFM Agreeableness). Our first aim was to examine change in personality traits related to social functioning and perceptions of social connectedness, as well as covariance between these sets of outcomes over time. We focused on Neuroticism, Extraversion, and Agreeableness in view of their relevance to social functioning. FFM Openness and Conscientiousness were also examined for comprehensiveness. In line with previous empirical and theoretical work (MacLean et al., 2011; Carhart-Harris et al., 2016b, 2021; Erritzoe et al., 2018; Forstmann et al., 2020; Netzband et al., 2020; Weiss et al., 2021) FFM Extraversion, Openness, Agreeableness, and perceived social connectedness were hypothesized to increase, and FFM Neuroticism was hypothesized to decrease following initial measurement.

Our second aim was to investigate factors that may potentiate or suppress personality change in relation to psychedelic experience. Few studies to date have rigorously examined potential moderators of change (e.g., subjective acute experiences, baseline personality) which may account for inconsistency in the literature and/or provide necessary conditions for change. Specifically, we examined the degree to which differences in FFM domains between timepoints varied as a function of an array of predisposing and acute factors. Predisposing factors relate to individual characteristics as well as psychological states, and spatial, temporal, and social contexts that may influence the nature of an individual’s experience. Acute factors relate to subjective psychological states arising under acute psychedelic effects. In line with previous work (MacLean et al., 2011; Erritzoe et al., 2018; Weiss et al., 2021), mystical-type experience was hypothesized to contribute to a larger difference in FFM Neuroticism, Extraversion, and Openness following 2weeks and 4weeks post-experience.

## Materials and Methods

### Study Design

This was a prospective study design using opportunity sampling and web-based data collection. The inclusion criteria were: >18years old, good comprehension of the English language, and having the intention to take a classic psychedelic drug (psilocybin/magic mushrooms/truffles, LSD/1P-LSD, ayahuasca, DMT/5-MeO-DMT, Salvia divinorum, mescaline, or iboga/ibogaine) in the near future. This approach provided the opportunity to collect a large amount of data in a non-controlled, naturalistic, and observational manner. The study consisted of a total of five surveys completed at different moments. The first survey was completed 1week before the planned psychedelic experience (Baseline); the second survey was completed 1day before the experience; the third survey was completed 1day after the psychedelic experience; and the fourth and fifth surveys were completed 2 and 4weeks following the experience.

### Participants

The following sample sizes were collected for each of the five surveys, respectively, N=654, N=535, N=379, N=315, and N=212. To better capture substantive effects of psychedelic use, 39 participants were eliminated who reported use of a low dose. An additional seven participants were eliminated who reported use of a substance without sufficiently strong serotonergic 2A receptor affinity [MDMA (4), ketamine (2), ibogaine (1)]. The final sample consisted of 148 volunteer participants who provided data at all timepoints at which dispositional trait-based measures were administered (i.e., 1week before experience, 2weeks post experience, 4weeks post experience). Because a larger number of participants reported on two timepoints versus all three, additional datasets were also used to examine change between baseline and 2weeks post (N=249) and baseline and 3weeks post (N=162). Unless otherwise noted, the N=148 sample was used for analyses. Demographic information is provided in Table 1. Information regarding the pharmacology of participants’ psychedelic experiences is provided in Table 2.

**Table 1:** Demographic Information.

Variable	Level	# (% of sample)/mean±SD
Gender	Male	104 (71%)
	Female	43 (29%)
	Other	1 (0%)
Age		31.5±11.9
Educational level	Left school before age 16 without qualifications	5 (3%)
	Some high school/GCSE level (in UK)	12 (8%)
	High school diploma/A-level education (in UK)	15 (10%)
	Some university (or equivalent)	26 (18%)
	Bachelor’s degree (or equivalent)	55 (37%)
	Post-graduate degree (e.g., Masters or Doctorate)	35 (24%)
Employment status	Student	42 (28%)
	Unemployed	15 (10%)
	Part-time job	25 (17%)
	Full-time job	61 (41%)
	Retired	5 (3%)
Nationality	United States	33 (22%)
	United Kingdom	34 (23%)
	Denmark	20 (14%)
	Germany	6 (4%)
	Canada	7 (5%)
	Other (28 in total)	38 (26%)
Psychiatric history	Lifetime diagnosis of psychiatric illness	48 (32%)
	Never diagnosed	100 (68%)
Previous psychedelic use	Never (psychedelic naïve)	13 (9%)
	Once	10 (7%)
	2–5 times	27 (16%)
	6–20 times	28 (17%)
	More than 20 times	30 (18%)

**Table 2:** Pharmacology of sample’s psychedelic experience.

		# (% of participants)
Compound type	LSD/1P-LSD	70 (47%)
	Psilocybin	34 (23%)
	Ayahuasca	17 (11%)
	DMT/5-MeO-DMT	5 (3%)
	Mescaline (Peyote, Huachuma/San Pedro)	6 (4%)
	Other	5 (3%)
Dose	A moderate dose	44 (30%)
	A high dose	63 (43%)
	A very high dose	19 (13%)
	An extremely high dose	11 (7%)
Psilocybin	1–1.99 grams	4
	2–2.99 grams	6
	3–3.99 grams	2
	5–5.5 grams	4
Truffles	1–15 grams	4
	15–29 grams	3
	30–45 grams	3
LSD/1P-LSD	60μg	1
	100–199μg	12
	200–299μg	23
	300–400μg	10
	800μg	1

Exact dosage information was not available for the majority of participants; therefore % of participants was not given for these levels; μg=micrograms; % of participants uses N=148 as the denominator.

Out of the total 741 participants who responded to any timepoint of the study, 576 (78%) dropped out or did not complete the first, fourth, or fifth survey. Because attrition can introduce sample bias and limit generalizability, analyses were conducted examining differences in study outcomes at baseline between the present sample and participants who did not complete the first, fourth, or fifth surveys. Results were indicative of significant differences (*p*<0.05) in one baseline personality item from the brief Ten-Item Personality Inventory (TIPI; Gosling et al., 2003). Completers exhibited lower TIPI Disorganized (i.e., higher Conscientiousness). However, this difference was not present when examining the larger datasets noted above (N=249 and N=262). Our results were consistent with previous work examining attrition-related sample bias in the present sample (Hübner et al., in press).^1^ As a note, the present sample has been used in previous work (Haijen et al., 2018; Roseman et al., 2019).

### Participant Recruitment and Dissemination of the Study

A website^2^ called “psychedelicsurvey.com” was created in collaboration with a team of web designers, and a specific domain was created for this specific study, which we called “global psychedelic survey” given the general nature of the sample and types of psychedelic use we were interested in capturing data on. This website contained all information needed for individuals to be informed about the study design, what was expected from participants and the informed consent. Once the informed consent was read and agreed on, individuals were able to sign up on the website by providing their name, email address and the date on which they expected to have their experience. Online advertisements, including a link to the main website that hosted the survey, were posted and shared on Facebook, Twitter, email newsletters, and online drug forums.^3^^,^^4^ Once participants signed up, they were included in an emailing system that was programmed to send out emails and reminders at specific times depending on the anticipated date of the psychedelic experience provided by the participants in the sign up process. Emails contained links to the relevant surveys, which were implemented and hosted by the online service system Survey Gizmo.^5^

### Ethical Considerations

The aim of the present study was to sample variables associated with psychedelic drug use, without manipulating or promoting such use. A disclaimer text on the website was included stating: “This survey should not be viewed as advocacy of psychedelic drug use. Its aim is to sample people whose intent to take a psychedelic is already established.” Participants provided their name and email address in the sign up process; however, this information was not saved in the survey responses nor used while handling the data. These data were only used to personalize the emails needed to send out the relevant survey links. When emails were sent out including the survey links, a unique identification code was generated which was included in the survey links as a uniform resource locator. This offered the opportunity to identify and link multiple survey responses of one individual without sampling privacy-sensitive information. Furthermore, Survey Gizmo has features that protect the security of responses, in line with the ethics requirements. The study was approved by Imperial College Research Ethics Committee and the Joint Research Compliance Office at Imperial College London.

### Measures

Unless otherwise noted, data for each measure were collected at the first, fourth, and fifth timepoints, and available for 148 participants.

#### Personality Outcomes

##### Ten-Item Personality Inventory

The TIPI (Gosling et al., 2003) consists of 10 items, with two items for each FFM domain: “Anxious, easily upset” and “calm, emotionally stable” index Neuroticism; “extraverted, enthusiastic,” and “reserved, quiet” index Extraversion; “critical, quarrelsome,” and “sympathetic, warm” index Agreeableness; “open to new experiences, complex” and “conventional, uncreative” index Openness; “dependable, self-disciplined” and “disorganized, careless” index Conscientiousness. Participants rated each item on a 7-point Likert-scale (1=Disagree strongly; 7=Agree strongly). Previous research has demonstrated good test–retest reliability and convergent validity for TIPI domains (Ehrhart et al., 2009). More broadly, FFM traits have shown adequate test–retest reliability across an average interval of 4weeks (*r*s>0.77; Gnambs, 2014), and longitudinal measurement invariance has been supported at the metric and scalar level in large samples (Lucas and Donnellan, 2011; Wortman et al., 2012). Unfortunately, consistent with past research (Gosling et al., 2003), internal consistency for three of five TIPI domains was low ([TIPI OpennessT1: *α*=0.40]; AgreeablenessT1: *α*=0.37; ConscientiousnessT1: α=0.48). Because heterogeneity in constructs can substantively obscure clear longitudinal changes and limit interpretation, we assessed TIPI items individually.

##### Empathic Concern and Perspective Taking

Two subscales from the Interpersonal Reactivity Index (IRI; Davis, 1983) were used to measure empathy and enrich measurement of FFM Agreeableness. IRI Empathic Concern is a 7-item subscale (e.g., “I am often quite touched by things that I see happen”) that measures affective empathy. Given high empirical overlap between IRI Empathic Concern and FFM Agreeableness, IRI Empathic Concern was considered to be reflective of Agreeableness (Mooradian et al., 2011; Melchers et al., 2016). More modern personality models have distinguished between two inter-related but distinct components of Agreeableness, namely Compassion and Politeness (DeYoung et al., 2007). Although no specific empirical work exists, IRI Empathic Concern is conceptually similar to BFAS Compassion (versus BFAS Politeness) and FFM facets of Tender-Mindedness, Altruism, and Trust (Judge et al., 2013). IRI Perspective Taking is a 7-item subscale (e.g., “I try to look at everybody’s side of a disagreement before I make a decision”) that measures cognitive empathy. Given moderate overlap between IRI Perspective Taking and FFM Agreeableness (Mooradian et al., 2011; Melchers et al., 2016) and the relevance of cognitive empathy to the present study, IRI Perspective Taking was also included in analyses, but was not considered directly reflective of Agreeableness. Participants rated each item on a 5-point Likert scale (1=Does not describe me well; 5=Describes me very well). Internal consistency (α) for IRI EC and IRI PT ranged from 0.81 to 0.85 across timepoints.

##### Compassion

The Santa Clara Brief Compassion Scale (SCBCS; Hwang et al., 2008) is a 5-item scale used to measure compassion. Items on the SCBCS were generated from Sprecher and Fehr’s (2005) Compassionate Love Scale which assesses altruistic love toward others. Given high empirical overlap between SCBCS and IRI Empathic Concern (Hwang et al., 2008), as well as theoretical and empirical relations between the construct of compassion and FFM Agreeableness (e.g., DeYoung, 2010; Judge et al., 2013), the SCBCS was treated as an index of the component of FFM Agreeableness involving Compassion (versus Politeness; Judge et al., 2013; DeYoung et al., 2007). Participants rated each item on a 7-point Likert-scale (1=Not at all true of me; 7=Very true of me). Internal consistency (α) ranged from 0.88. to 0.90 across timepoints.

##### Tellegen Absorption

The Tellegen Absorption Scale (MODTAS; Tellegen et al., 1991) is used to measure trait absorption. The etymology of absorption is rooted in the search for reliable personality correlates of hypnotizability (de Groh, 1989), but it soon became clear to scholars that absorption also bore high theoretical and empirical overlap with FFM Openness (e.g., Glisky et al., 1991). This work indicated that Absorption strongly overlaps with and therefore presents a good index of certain components of Openness (but not others), including aesthetic appreciation, fantasy, unusual associations, unconventional worldviews, and awareness of inner feelings; Absorption was not observed to overlap with intellectual curiosity, openness to unusual ideas, or liberal values. For brevity, only the 25 scored items were included in this survey. Participants rated each Absorption item on a 5-point Likert-scale (1=Never, 5=Very often). Internal consistency (α) ranged from 0.92 to 0.93 across timepoints.

#### Social Connectedness Outcomes

##### Social Connectedness

The Social Connectedness Scale (SCS; Lee and Robbins, 1995) is an 8-item scale that measures one’s perceived sense of belongingness in relation to others and society (e.g., “Even around people I know, I do not feel that I really belong”). Participants rated each item on a 6-point Likert scale (1=Strongly disagree; 6=Strongly agree). Scores were reverse-scored such that higher scores indicated greater social connectedness. Internal consistency (α) ranged from 0.94 to 0.95 across timepoints.

##### Relatedness

The Relatedness scale is a graphical scale that measures one’s perceived sense of relationship with other human beings. The measure uses a 7-item Likert scale consisting of two circles (“Self,” “Other”) that progressively overlap (see Supplementary Figure S1). The scale has been used in previous work (Forstmann et al., 2020) and is a modified version of the “inclusion of others in the self” scale which assesses how strongly people include their romantic partners in their self-construal (Aron et al., 1991). Participants were asked to select the image that best describes their “current relationship with other human beings, in general.”

### Moderators

#### Evaluation of Validity

##### Suggestibility

The Multidisciplinary Iowa Suggestibility Scale-Short (MISS; Kotov et al., 2004) was used to measure participants’ susceptibility to internalize external influences. The MISS is a 21-item self-report scale that uses a 5-point Likert-scale (1=Not at all; 5=A lot). Internal consistency (α) was 0.85. Data were collected in the first survey.

##### Expectancy

Expectancy of favorable change was measured using one original item (“How confident are you that the upcoming psychedelic experience will have a long-lasting positive effect?”). Data were collected in the first survey.

#### Predisposing Factors

##### Set and Setting

An original 12-item measure was constructed to assess participants’ cognitive and emotional relationship to their psychedelic experience to come. Previous factor-analytic work derived three parsimonious factors: Set (describing openness toward and psychological preparedness for the experience; 7 items; *α*=0.75), Setting (describing positive feelings toward experience companions and planned environment; 3 items; *α*=0.74), and Clear Intentions (describing strong and clear expectations; 2 items; *α*=0.46). Participants rated each item on a visual analogue scale ranging from 0 (Strongly disagree) to 100 (Strongly agree). Data were collected in the second survey, and were available for 145 participants.

##### Motives

An original 10-item measure was constructed to assess the types of experiences participants sought to consciously create. Previous factor-analytic work derived three parsimonious factors: Spiritual connection (describing motivation for spiritual, religious, nature-based, or growth-oriented experience; 4 items; *α*=0.56), Recreation (describing motivation for social, fun, explorative experience; 3 items; *α*=0.37), and Emotional (describing motivation for addressing difficult emotions; 3 items; *α*=0.48). Participants rated each item using a 4-point Likert scale (1=Not at all; 4=Very much). Data were collected in the first survey.

##### Baseline Anxiety

A 6-item short version of the Spielberger State–Trait Anxiety Inventory (STAI-SF; Marteau and Bekker, 2020) was used to measure trait anxiety. Participants rated each item on a 4-point Likert scale (1=Not at all; 4=Very much). Internal consistency (α) was 0.85. Data were collected in the first survey.

##### Baseline Depression

The Quick Inventory of Depressive Symptoms-Self-Report-16 (QIDS; Rush et al., 2018) is a 16-item scale that measures severity of depressive symptoms within the preceding 7days. Participants rated each item on a 4-point Likert scale whose content varies by item. The QIDS consisted of 9 subscales comprising one to four items. For subscales involving multiple items, the maximum score across the items was assigned. The QIDS total score represented the mean of the 9 subscales. Internal consistency (α) across these nine QIDS subscales was 0.81. Data were collected in the first survey.

#### Acute Factors

The following measures were sent to participants 1day after they were due to have their psychedelic experience (third survey). Data for these measures were available for 137 participants.

##### Challenging Experience

The Challenging Experience Questionnaire (CEQ; Barrett et al., 2016) is a 26-item scale that measures unpleasant affective, cognitive, and somatic reactions to psychedelic compounds. Reactions include isolation, grief, physical distress, fear, insanity, paranoia, and death (e.g., “I had the feeling something horrible would happen”). The CEQ is derived from “challenging” items from other psychedelic questionnaires: Hallucinogen Rating Scale (HRS) (Strassman et al., 1994), Altered State of Consciousness questionnaire (Dittrich, 1998), and States of Consciousness Questionnaire (Pahnke et al., 1969; Griffiths et al., 2006). Participants rated each item on a 6-point Likert-scale (1=None, not at all; 5=Extreme, more than any other time in my life). Internal consistency (α) was 0.95.

##### Emotional Breakthrough

The Emotional Breakthrough Inventory (EBI; Roseman et al., 2019) is a 6-item scale that measures productive engagement with emotional problems (e.g., “I felt able to explore challenging emotions and memories”). Participants rated each item on a visual analogue scale ranging from 0 (No, not more than usually) to 100 (Yes, entirely or completely). Internal consistency (α) was 0.93.

##### Mystical Experience

The Mystical Experience Questionnaire (MEQ; MacLean et al., 2012; Barrett et al., 2015) is a 30-item scale that measures mystical aspects of participants’ experiences. The MEQ’s items were originally represented on the Pahnke-Richards MEQ (Pahnke, 1969; Richards, 1975). In line with psychometric work (Barrett et al., 2015), four subscales were assessed: Mystical (15-item; e.g., “Experience of the fusion of your personal self into a larger whole”), Positive mood (6-item; e.g., “Sense of awe or awesomeness”), Transcendence of time and space (6-item; e.g., “Loss of your usual sense of space”), and Ineffability (3-item; e.g., “Sense that the experience cannot be described adequately in words”). Participants rated each item on a 6-point Likert scale (1=None, Not at all; 6=Extreme, more than any other time in your life and stronger than 5). Internal consistency (α) ranged from 0.89 (Time Space) to 0.96 (Mystical).

##### Dosage

One original item was used to measure approximate dosage of participants’ use. Participants rated their dose on a 5-point response scale [1=A low dose (equivalent to 50 micrograms LSD); 5=Extremely high dose (equivalent to 300 micrograms LSD)]. Data were collected in survey three.

### Analytic Plan

#### Analyses

Four sets of analyses were planned. To reduce Type I error, across analyses, a statistical significance threshold was set at *p*<0.005 (Benjamin et al., 2018), whereas *p*<0.01 was set for hypotheses. The first set of analyses examined the degree to which personality and perceived social connectedness outcomes changed in relation to psychedelic experience. Linear mixed models were conducted (equivalent to one-way repeated measures ANOVA) to determine the persisting effects of psychedelic compounds on outcomes, comparing each outcome between each timepoint (baseline, 2weeks post, and 4weeks post). The full N=148 dataset was principally used, containing participants who reported on outcomes at all timepoints. However, to maximize sample size and power, additional models were conducted in larger datasets to examine change between baseline and 2weeks post (N=249) and baseline and 4weeks post (N=162). Where the significance of results from the larger datasets contradicted results from the smaller (N=148) dataset, results from the larger dataset were presented. The following indices of effect size were used: Unstandardized (B) coefficients indicate mean differences between timepoints. *dz* indicates effect size change in outcome scores in terms of the standard deviation of within-subject change scores (e.g., T2-T1; Lakens, 2013). Cohen’s *ds* (standard *Cohen’s d*; Cohen, 1988) effect size estimates were calculated using the following equation: (Mean-score _T2_-Mean-score _T1_)/((SD_T1_)^2^+SD_T2_)^2^)^0.5^. Marginal R^2^ values were used to indicate the degree to which the fixed effect of Time accounted for variance in outcomes.

The second set of analyses used linear mixed models to examine moderating effects on time. Linear mixed effects models were conducted in which we included moderators as fixed covariates to the base model (including Time). Three sets of moderator variables were examined including validity variables (including biases, expectancy, suggestibility), predisposing factors (including demographic variables, lifetime use of psychedelics, motives, baseline traits), and acute factors. For continuous moderators, unstandardized (*B*) coefficients were used to indicate the added effect of the moderator to the effect of time at one standard deviation above the mean of the moderator. For dichotomous moderators, unstandardized (*B*) coefficients were used to indicate the added effect of the moderator to the effect of time at one level of the moderator versus the other. Marginal R^2^ values were used to indicate the degree to which fixed effect variables (i.e., Time, moderator) accounted for variance in outcomes.

The third set of analyses used regression to examine covariation between personality and perceived social connectedness outcomes over time. Change scores for each outcome were calculated by subtracting timepoint one (baseline) scores from timepoint three (4weeks post), and correlations were subsequently calculated between each personality outcome and each social connectedness outcome. The fourth set of analyses calculated zero-order correlations among baseline outcomes (Supplementary Table S1).

#### Power Analyses

*Post hoc* power analyses (using “simr” package in R) were conducted to assess power for the first and second sets of analyses. For each set of analyses, effect sizes sufficient to obtain 80% statistical power [alpha value *p*=0.005 (*p*=0.01 for hypotheses), using 100 Monte Carlo simulations] were estimated. Results indicated that the self-report sample was powered (80%) to accurately detect true differences between timepoints exceeding 0.10/0.12 (Tellegen Absorption), 0.12/0.14 (IRI Perspective Taking), 0.12/0.16 (IRI Empathic Concern), 0.13/0.15 (SCBCS Compassion), 0.15/0.19 (TIPI Reserved), 0.17/0.19 (TIPI Extraverted, TIPI Anxious), 0.17/0.21 (TIPI Calm), 0.17/0.24 (TIPI Disorganized), 0.18/0.20 (TIPI Sympathetic), 0.18/0.21 (TIPI Critical), 0.19/0.19 (SCS Social Connectedness), 0.19/0.23 (Relatedness, TIPI Conventional), 0.22/0.25 (TIPI Dependable) and 0.25/0.30 (TIPI Open-minded) standard deviations. The first value reflects power within the N=249 sample (containing baseline and 2weeks timepoints) and the second value reflects power within the N=162 sample (containing baseline and 4weeks timepoints). With respect to moderation-based analyses, analyses were powered (80%) to detect true interaction effects of small to medium size (standardized interaction coefficient effect size ranged from 0.13 to 0.30 across domains and moderators).

## Results

### Examining Change in Personality and Social Connectedness

**Figure 1:**
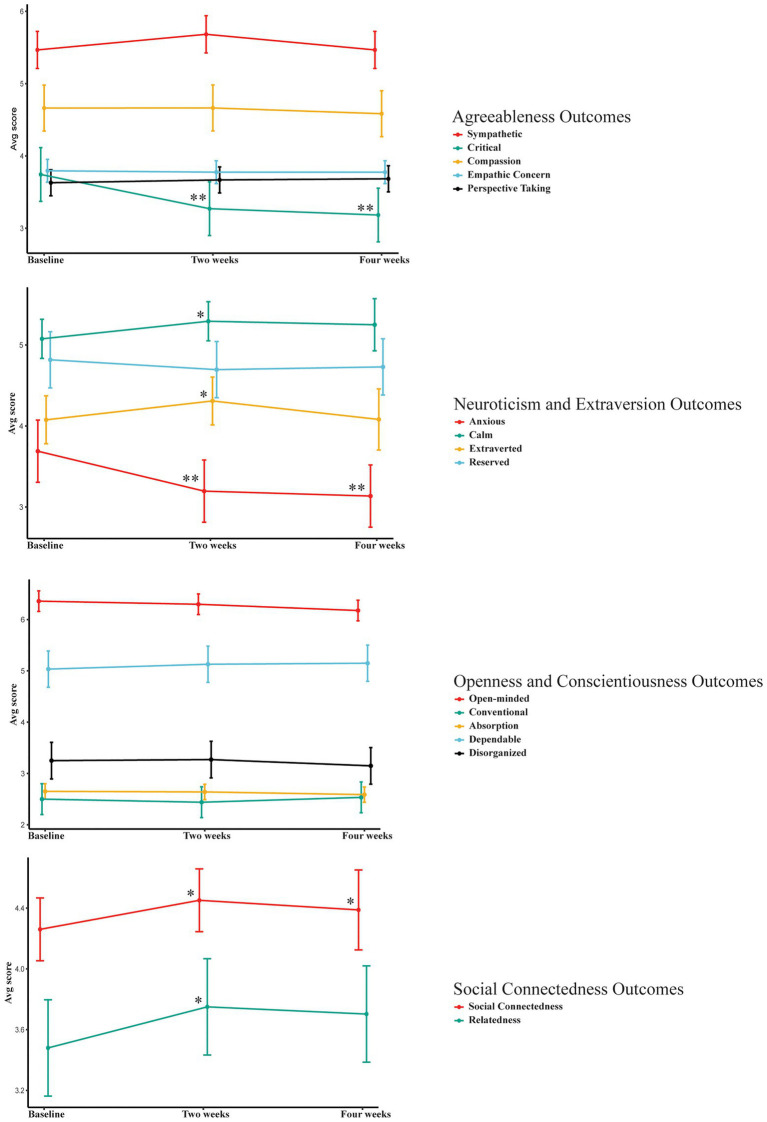
Line plots illustrating change in outcomes. Error bars represent 99% confidence intervals around means for hypothesized outcomes including Agreeableness, Neuroticism, Extraversion, and Social Connectedness; and 99.5% confidence intervals around means for Conscientiousness and Perspective Taking. First and second sets of asterisks denote a significant difference between baseline and 2weeks and 4weeks, respectively, following psychedelic experience. ^*^p<0.01; ^**^p<0.005.

Results demonstrated that 2weeks following psychedelic experience, TIPI Anxious (B=-0.49, *p*<0.0001) and TIPI Critical (B=−0.47, *p*<0.0001) were significantly lower; and TIPI Calm (B=0.22, *p*=0.006), TIPI Extraverted (B=0.23, *p*=0.009), Social Connectedness (B=0.19, *p*=0.008), and Relatedness (B=0.27, *p*=0.006) were significantly higher. Four weeks following psychedelic experience, TIPI Anxious (B=−0.55, *p*<0.0001) and TIPI Critical (B=−0.56, *p*<0.0001) remained significantly lower; and Social Connectedness (B=0.18, *p*=0.008) remained significantly higher. These results are shown in Figure 1, and expanded upon in Table 3 where 99% confidence intervals and effect size values (i.e., *dz* and Cohen’s *ds*) are provided. Full results can be found in Supplementary Table S2.

**Table 3:** Examining significant main effects of time.

Timepoint comparison	Outcome	B	99% CI	*dz*	*ds*
Baseline & 2weeks post	TIPI Critical	−0.47^**^	−0.18,−0.77	−0.34	−0.19
Baseline & 4weeks post	TIPI Critical	−0.56^**^	−0.26,−0.86	−0.39	−0.23
Baseline & 2weeks post	TIPI Anxious	−0.49^**^	−0.20, −0.78	−0.37	−0.19
Baseline & 4weeks post	TIPI Anxious	−0.55^**^	−0.27−0.84	−0.39	−0.22
Baseline & 2weeks post	TIPI Calm	0.22^*^	0.01,0.42	0.18	0.10
Baseline & 2weeks post	TIPI Extraverted	0.23^*^	0.00,0.46	0.17	0.09
Baseline & 2weeks post	Social Connectedness Scale	0.19^*^	0.01,0.37	0.17	0.11
Baseline & 4weeks post	Social Connectedness Scale	0.18^*^	0.01,0.36	0.21	0.10
Baseline & 2weeks post	Relatedness	0.27^*^	0.02,0.52	0.26	0.15

TIPI, Ten-Item Personality Inventory; CI, Confidence Interval; Unstandardized (B) coefficients indicate mean differences between timepoints. *dz* indicates effect size change in outcome scores in terms of the standard deviation of within-subject change scores (e.g., T2-T1; Lakens, 2013). Cohen’s ds (standard Cohen’s d; Cohen, 1988) effect size estimates were calculated using the following equation: (mean-score T2-mean-score T1)/((SDT1)2+SDT2)2)0.5. ^*^p<0.01; ^**^p<0.005.

### Examining Moderation of Change

To investigate the degree to which change between timepoints depended on predisposing and acute factors, moderation by three sets of variables were examined including validity variables (including biases, expectancy, suggestibility), predisposing factors (including demographic variables, lifetime use of psychedelics, motives, baseline traits), and acute factors. Each of these sets will be examined in turn below. Tables 4 and 5 are provided to show instances of significant moderation of differences between at minimum two timepoints, and to present change in marginal R^2^ after adding interaction terms. Full results of significant moderation-based models are provided in Supplementary Table S2.

**Table 4:** Incremental variance explained by moderators.

Moderator	Neuroticism		Extraversion		Agreeableness		Empathic	Perspective	Santa Clara	Social	Relatedness
	Anxious	Calm	Extra	Reserved	Critical	Sympath	Concern	Taking	Compassion	Connectedness
**Validity**											
Pro-use Bias	0.001	0.001	0.012	0.004	0.001	0.013	0.039	0.035	0.036	0.015	0.045
Therapy Bias	0.000	0.001	0.018	0.002	0.003	0.025	0.074	0.045	0.090	0.024	0.058
Knowledge Bias	0.014	***0.022***	0.003	0.000	0.001	0.016	0.018	0.049	0.003	***0.029***	0.002
Experienced Bias	0.085	***0.067***	0.031	0.004	0.000	0.006	0.024	0.061	0.026	0.041	0.032
Expectancy	0.079	0.069	0.040	0.012	0.036	0.055	0.072	0.116	0.074	0.058	0.061
Suggestibility	0.019	0.013	0.004	0.003	0.033	0.012	0.003	0.003	0.003	0.000	0.020
**Predisposing Factors**											
Age	0.059	0.034	0.004	0.002	0.037	0.006	0.012	0.003	0.002	0.006	0.005
Education Level	0.025	0.008	0.011	0.001	0.016	0.007	0.090	0.062	0.059	0.048	0.001
Sex	0.018	0.005	0.003	0.004	0.030	0.048	0.036	0.014	0.023	0.014	0.001
Psychedelic Naive	0.008	0.000	0.009	0.006	0.002	0.001	0.000	0.009	0.002	0.004	0.003
Psychedelic Uses	0.070	0.029	0.034	0.007	0.004	0.003	0.027	***0.029***	0.029	0.022	0.008
Set	0.082	0.071	0.031	0.017	0.002	0.017	0.007	0.005	0.003	0.042	0.007
Setting	0.028	0.074	0.015	0.008	0.007	0.015	0.010	0.007	0.018	0.038	0.023
Clear intentions	0.006	0.010	0.002	0.004	0.001	0.002	0.033	0.025	0.025	0.024	0.006
Spiritual motive	0.036	0.032	0.090	0.039	0.005	0.026	0.085	0.052	0.088	0.019	0.063
Recreation Motive	0.011	0.002	0.005	0.003	0.017	0.010	0.014	0.000	0.023	0.002	0.007
Emotional Motive	0.056	0.038	0.014	0.021	0.002	0.002	0.001	0.001	0.004	0.023	0.001
Respective Baseline Trait	***0.677***	***0.688***	0.704	0.684	0.621	0.624	0.829	***0.818***	0.808	0.640	***0.613***
Baseline STAIT Anxiety	0.382	0.325	0.051	0.004	0.024	0.021	0.005	0.031	0.005	0.145	0.031
Baseline QIDS Depression	0.289	0.203	0.054	0.012	0.006	0.014	0.012	0.019	0.005	0.156	0.025
**Acute Factors**											
Challenging Exp	0.034	0.046	0.028	0.023	0.002	0.006	0.003	0.002	0.005	0.022	0.005
Emo Breakthrough	0.018	0.004	0.002	***0.007***	0.007	0.008	0.029	0.021	0.021	0.004	0.011
Mystical	0.023	0.026	0.026	0.012	0.003	0.036	0.101	0.140	0.084	0.029	0.048
Positive Mood	0.025	0.040	0.031	0.016	0.006	0.020	0.056	0.161	0.048	0.024	0.019
Time–space	0.005	0.004	0.009	0.004	0.001	0.020	0.009	0.025	0.007	0.009	0.021
Ineffability	0.003	0.008	0.001	0.001	0.022	0.002	0.001	0.007	0.006	0.002	0.004
Dosage	0.001	0.022	0.014	0.017	0.001	0.001	0.016	0.011	0.014	0.016	0.021

Bolded/italicized marginal R^2^ values are indicative of instances of significant moderation at *p*<0.005 threshold. Italicized marginal R^2^ values for Respective Baseline Trait moderators denote a significant interaction term that is significant at *p*<0.005 threshold and survived tests of regression to the mean. Marginal R^2^ values indicate the degree to which fixed effect variables (e.g., Time, moderator) account for variance in outcomes. Extra=Extraverted; Sympath=Sympathetic.

**Table 5:** Incremental variance explained in outcomes related to openness and conscientiousness by moderators.

Moderator	Openness		Tellegen	Conscientiousness	
	Open-minded	Conventional	Absorption	Dependable	Disorganized
**Validity**					
Pro-use Bias	0.018	0.005	0.005	0.004	0.017
Therapy Bias	0.002	0.005	0.020	0.009	0.006
Knowledge Bias	0.008	0.065	0.026	0.019	0.004
Experienced Bias	0.048	0.023	0.023	0.031	0.008
Expectancy	0.030	0.037	0.086	0.046	0.010
Suggestibility	0.005	0.061	0.018	0.041	0.032
**Predisposing Factors**					
Age	0.008	0.017	0.000	0.008	0.072
Education Level	0.020	0.027	0.001	0.001	0.008
Sex	0.010	0.023	0.029	0.003	0.008
Psychedelic Naive	0.005	0.008	0.007	0.000	0.029
Psychedelic Uses	0.028	0.013	0.023	0.008	0.003
Set	0.023	0.014	0.003	0.031	0.003
Setting	0.035	0.042	0.020	0.047	0.041
Clear Intentions	0.003	0.001	0.000	0.007	0.049
Spiritual Motive	0.028	0.045	0.145	0.013	0.000
Recreation Motive	0.005	0.016	0.000	0.004	0.012
Emotional Motive	0.002	0.005	0.006	0.034	0.007
Respective Baseline Trait	0.366	0.596	0.855	0.569	0.654
Baseline STAIT Anxiety	0.028	0.006	0.004	0.066	0.013
Baseline QIDS Depression	0.018	0.008	0.012	0.089	0.058
**Acute Factors**					
Challenging Exp	0.015	0.012	0.045	0.001	0.005
Emo Breakthrough	0.020	0.059	0.057	0.003	0.007
Mystical	0.008	0.058	0.134	0.011	0.006
Positive Mood	0.002	0.016	0.034	0.010	0.002
Time–space	0.001	0.030	0.065	0.006	0.002
Ineffability	0.002	0.008	0.000	0.004	0.001
Dosage	0.004	0.001	0.009	0.008	0.033

Bolded/italicized marginal R^2^ values are indicative of instances of significant moderation at *p*<0.005 threshold. Italicized marginal R^2^ values for Respective Baseline Trait moderators denote a significant interaction term that is significant at *p*<0.005 threshold and survived tests of regression to the mean. Marginal R^2^ values indicate the degree to which fixed effect variables (e.g., Time, moderator) account for variance in outcomes.

#### Validity Variables

With respect to validity variables, three types of variables were examined: biases, expectancies of change, and suggestibility. First, biases involving favorable attitudes toward psychedelic use (e.g., “I have an advanced knowledge about psychedelics”) did not contribute to overreporting of change, as intended in study design, but rather showed evidence of suppressing change in TIPI Calm and Social Connectedness. Specifically, participants endorsing advanced knowledge of psychedelics (Knowledge Bias) and high experience with psychedelic use (Experience Bias) exhibited higher baseline TIPI Calm, and showed no change in TIPI Calm at 2weeks or 4weeks, whereas participants denying advanced knowledge and high experience showed a greater increase in TIPI Calm at 2weeks (*B*=0.28, *B*=0.24, respectively) and 4weeks (*B*=0.29, *B*=0.34, respectively). For example, a one-standard-deviation decrease in self-reported “high psychedelic experience” was associated with an incremental 0.23 unit increase in TIPI Calm (on 7-point Likert scale) following 2weeks and an incremental 0.34 unit increase following 4weeks. Similarly, participants denying advanced knowledge of psychedelics showed a greater increase in Social Connectedness following 2weeks (*B*=0.23), whereas participants endorsing advanced knowledge showed no change. That is, a one-standard-deviation decrease in self-reported “advanced knowledge” was associated with an incremental 0.23 unit increase in SCS (on 6-point Likert scale) following 2weeks.

#### Predisposing Factors

With respect to predisposing factors, five sets of variables were examined: demographic variables, lifetime psychedelic uses, set and setting, motives, and baseline traits. Lifetime psychedelic uses and baseline traits emerged as significant moderators.

With respect to lifetime psychedelic uses, a one-standard-deviation decrease in lifetime psychedelic uses amplified increases in IRI Perspective Taking by 0.11 and 0.09units (on 5-point Likert scale) two and 4weeks following psychedelic experience, respectively. Specifically, participants endorsing average to high frequency of previous psychedelic use showed no change in Perspective Taking, whereas participants endorsing low (~one-standard-deviation below mean) previous psychedelic uses exhibited significant increases in Perspective Taking following two and 4weeks (*B*_T1-T2_=0.11, *p*=0.002; *B*_T1-T3_=0.09, *p*=0.005).

With respect to baseline traits, initial analyses were indicative of moderation by baseline traits in the cases of 14 of 16 outcomes. However, because regression to the mean is likely responsible for many of these trends in adaptive directions (e.g., participants high in baseline anxiety exhibiting amplified decreases in anxiety), subsequent tests were conducted. Effect size estimates were examined while excluding participants with extreme baseline scores most conducive to regression in an adaptive trait direction, namely above the 80th quantile of baseline TIPI Anxious, STAIT Anxiety, QIDS Depression, TIPI Reserved, TIPI Conventional, TIPI Critical, and TIPI Disorganized, and below the 20th quantile of the other outcomes. Next, moderation-based results were inspected using linear mixed models, plots, and pairwise contrasts. An adaptive moderation effect was deemed credible where (a) the interaction term remained statistically significant (*p*<0.005) and in the direction of an adaptive effect; and (b) significant (*p*<0.005) changes in an adaptive direction were present among participants at the maladaptive level of the moderator (e.g., where participants one standard deviation above the mean of baseline anxiety showed significant decreases in anxiety). Only moderated change between baseline and 4weeks post is reported and interpreted. Adaptive moderation effects that survived these tests of regression to the mean were found for TIPI Anxious, TIPI Calm, IRI Perspective Taking, and Relatedness (see Supplementary Table S1 for detailed results). Using the full dataset, pairwise contrasts were calculated to examine temporal changes in outcomes at different levels of these baseline traits. Participants who presented to the study with high (i.e., ~ one-standard-deviation above mean) baseline TIPI Anxious exhibited statistically significant reductions in these items following 4weeks (*B*_T1-T3_=-0.75, *p*<0.0001). Similarly, participants with low (~one-standard-deviation below mean) baseline TIPI Calm (*B*_T1-T3_=0.67, *p*<0.0001), IRI Perspective Taking (*B*_T1-T3_=0.21, *p*<0.0001), and Relatedness (*B*T1-T3=0.63, *p*<0.0001) exhibited significant increases in these items following 2 and 4 weeks. Notably, TIPI Anxious was the only outcome for which adaptive change was also found among participants who exhibited average baseline scores. These results are, however, considered tentative given the likely influence of regression to the mean.

#### Acute Factors

With respect to acute factors, we examined moderation of temporal change in outcomes by Challenging Experience, Emotional Breakthrough experience, and mystical-type experience including MEQ Mystical, Positive Mood, Space Time, and Ineffability. Dosage was also examined in view of empirical effects on self-reported acute intensity, personal meaning, and spiritual significance (e.g., Griffiths et al., 2011). Only Emotional Breakthrough experience emerged as a significant moderator. Specifically, a one-standard-deviation increase in self-reported Emotional Breakthrough amplified decreases in TIPI Reserved by 0.34units (on 7-point Likert scale) 4 weeks following psychedelic experience. Results indicated that a significant decrease in TIPI Reserved depended on participants’ experience of emotional breakthrough, such that only participants who exhibited a one-standard-deviation increase in Emotional Breakthrough showed a meaningful change (decrease) in TIPI Reserved. Because Emotional Breakthrough did not evince an influence on other personality outcomes including the other Extraversion-based TIPI item, nor did it exhibit an effect on TIPI Reserved between Baseline and 2weeks post, it is possible that this effect is the product of Type I error, and as such, should be interpreted with caution.

### Examining Covariation Between Outcomes Over Time

#### Personality and Social Connectedness

To determine whether changes in outcomes covaried over time with other outcomes, we computed change scores between baseline and 4weeks following psychedelic experience, and calculated the correlations between these change scores (Table 6). The N=162 sample was used. Two sets of analyses were conducted. First, we examined the degree to which each personality outcome covaried over time with Social Connectedness and Relatedness. Results indicated that Social Connectedness showed significant covariation over time with TIPI Anxious (*r*=−0.23, *p*=0.003), TIPI Extraverted (*r*=0.27, *p*<0.0001), TIPI Critical (*r*=−0.22, *p*=0.004), and TIPI Disorganized (*r*=−0.26, *p*=0.001). Second, we examined the degree to which personality outcomes covaried over time with each other. In this set, only personality traits that exhibited significant main effect change from previous analyses were examined. Results indicated significant covariation between TIPI Anxious and TIPI Critical (*r*=−0.29, *p*<0.0001) over time.

**Table 6:** Examining covariation over time (baseline—four weeks) between personality and social connectedness outcomes using correlations between change scores.

ΔPersonality outcome	ΔSocial connectedness	ΔRelatedness
ΔTIPI Anxious	−0.23^**^	−0.07
ΔTIPI Calm	0.14	0.07
ΔTIPI Extraverted	0.27^**^	0.12
ΔTIPI Reserved	0.14	0.07
ΔTIPI Critical	−0.22^**^	−0.02
ΔTIPI Sympathetic	0.22^*^	0.17ʹ
ΔIRI Empathic Concern	0.03	0.05
ΔIRI Perspective Taking	0.10	0.17ʹ
ΔSanta Clara Compassion	0.14	0.16ʹ
ΔTIPI Open-minded	0.05	0.07
ΔTIPI Conventional	−0.12	−0.02
ΔTellegen Absorption	0.03	0.17ʹ
ΔTIPI Dependable	−0.06	−0.05
ΔTIPI Disorganized	−0.26^**^	0.00

*p<0.05;* ^*^p<0.01; ^**^p<0.005.

## Discussion

Meaningful prosocial changes were observed across a number of outcomes relevant to social functioning. Overall, participants reported decreases in critical and quarrelsome demeanor and anxiety and mood lability, and increases in perceived social connectedness. Preliminary evidence was additionally found for increases in cognitive empathy, but only among participants initially low in this trait. Adaptive changes in neuroticism and perceived social connectedness also showed signs of being amplified among participants starting with less adaptive initial levels of these traits. We expand upon these findings within the context of four main questions: (a) Is psychedelic use related to changes in personality traits relevant to social functioning? (b) Is psychedelic use related to change in perceptions of social connectedness? (c) Is change in personality connected to change in perceived social connectedness? and (d) Are there factors that predispose or potentiate change in social functioning-related traits and perceived social connectedness?

### Is Psychedelic Use Related to Changes in Personality Traits Relevant to Social Functioning?

Two personality domains with strong relevance to social functioning (neuroticism, agreeableness) displayed substantive, small-sized, changes in the direction of enhanced social functioning. First, agreeableness was measured using four outcomes including TIPI Critical Quarrelsome, TIPI Sympathetic Warm, compassion, and empathic concern. TIPI Critical Quarrelsome was the only outcome to decline substantively between baseline measurement and 2weeks following psychedelic experience and remain substantively below baseline scores 4weeks following use. Although limited internal reliability for this single-item outcome qualifies interpretation, this result is suggestive of components of agreeableness that are particularly susceptible to psychedelic change processes. According to the Big Five Aspects model (BFAS; DeYoung et al., 2007), a data-driven model of personality, each FFM domain can be divided into two distinct, but interrelated, components that hold superior parsimony relative to FFM facets, and greater granularity relative to broad FFM domains. For agreeableness, these components represent aspects Compassion (versus Callousness) and Politeness (versus Belligerence). Notably, TIPI Critical Quarrelsome is the only measured outcome that, based on rational analysis, conceptually captures the Politeness aspect of agreeableness, whereas the other agreeableness outcomes capture the Compassion aspect. At its low pole, Politeness, like the TIPI Critical Quarrelsome item, describes an antagonistic and conflict-prone style of interpersonal relating that seeks to express judgment, demonstrate superiority, and gain advantage.

Our results are the first in the literature to suggest a specific decrease in criticism and quarrelsomeness following psychedelic experience. Should psychedelic experience be most targeted to personality functioning in Politeness, it bears noting the clinical and forensic implications of this finding. Politeness, relative to Compassion, bears strongest relations with pathological personality traits most relevant to antagonistic personality disorders (e.g., Narcissistic Personality Disorder, Antisocial Personality Disorder) including attention seeking, grandiosity, manipulativeness, deceitfulness, and hostility (e.g., DeYoung et al., 2016). As such, in this data, psychedelic use shows some signs of therapeutic potential for the treatment of personality disorders. Our data appears consistent with three studies that have shown qualified support for prospective increases in agreeableness (Carhart-Harris et al., 2016b; Netzband et al., 2020; Weiss et al., 2021), as well as work showing decreased levels of supervision failure among individuals involved in the justice system and intimate partner violence in the general population (Hendricks et al., 2014; Thiessen et al., 2018).

No other agreeableness outcomes exhibited meaningful change. One explanation for these results relates to restriction of range in these outcomes versus TIPI Critical Quarrelsome, such that participants in our sample tended to exhibit already above-average standing on TIPI Sympathetic Warm, compassion, and empathic concern, and thus may have possessed less potential for upward change in these traits (see Supplementary Figure S2 for baseline density plots). Moderation analyses were accordingly conducted to examine whether participants with lower scores on these traits were more likely to exhibit significant upward change. No credible moderation was observed. Power analyses were additionally conducted to evaluate our capacity to form conclusions from these null results, namely that TIPI Sympathetic Warm, compassion, and empathic concern do not meaningfully respond to naturalistic psychedelic use. The results of our power analyses were suggestive that we were adequately powered to detect an interaction effect of small size (*b*<0.20) for compassion and empathic concern if one was present. As such, our data provide preliminary support for the absence of meaningful longer-term change in compassion and empathic concern in the general population of psychedelic users. Furthermore, our null results with respect to adaptive change in affective empathy (indexed by IRI Empathic Concern) are convergent with previous evidence that explicit emotional empathy (a related construct) does not remain enhanced for longer than 1week following psychedelic administration (Mason et al., 2019).^6^ It is notable that acute increases in explicit emotional empathy have been observed in three previous studies using the Multifaceted Empathy Test (MET; Dziobek et al., 2008) (Dolder et al., 2016; Pokorny et al., 2017; Mason et al., 2019). This raises the possibility that enhancements in affective empathy and compassion may be time-limited without additional maintenance practices.

Finally, perspective taking, a related construct capturing cognitive empathy (i.e., ability to occupy mental perspectives of others; IRI Perspective Taking), did show some signs of increasing in relation to psychedelic use for a subset of our sample. Although a main effect change in perspective taking was not observed, participants one standard deviation below the mean in baseline perspective taking exhibited substantive change in this trait. Although the measure we used involved subjective self-appraisal, our results provide preliminary evidence that psychedelic use may provide longer-term enhancements to cognitive empathy among individuals with low initial capacity. Of note, however, Mason et al. (2019) did not observe enhanced cognitive empathy 7days post-psychedelic experience while using the MET cognitive task. We encourage researchers to examine whether a similar subset of individuals with low standing on cognitive empathy exhibit substantive post-acute enhancements in objective task performance.

Collectively, this pattern of results may be suggestive that psychedelic experience in the general population is more likely to drive adaptive changes in Politeness versus Compassion. On a methodological level, the pattern raises the possibility that mixed findings within the literature may owe in part to imprecise measurement of agreeableness. Future research is accordingly called for that examines psychedelic-induced personality change using granular measurement sufficient to detecting possible differential effects on aspects (or facets) of personality.

Second, perhaps strongest support was found for adaptive change in neuroticism, with data showing small-sized, but substantive decreases in anxiety and mood lability (TIPI Anxious Easily upset) that remained significantly above baseline scores 4weeks following psychedelic use. Our results are consistent with a number of previous prospective studies showing psychedelic-induced reductions in neuroticism (Barbosa et al., 2009; Fernández et al., 2014; Erritzoe et al., 2018) including one study demonstrating change relative to a control group (Netzband et al., 2020) and a naturalistic study showing change in self- and informant-report data (Weiss et al., 2021). Three randomized and controlled clinical trials (Palhano-Fontes et al., 2019; Davis et al., 2020; Carhart-Harris et al., 2021) also provide support for a psychedelic-induced antidepressant effect, which bears relevance to our present examination of neuroticism in view of common structure between personality and psychopathology (e.g., Widiger et al., 2012; Kotov et al., 2017). Of note, the size of the observed effects are somewhat smaller than medium-sized effects found in other studies (e.g., Erritzoe et al., 2018; Weiss et al., 2021). As such, recreational use outside of therapeutic contexts designed to support introspective inquiry (e.g., psychedelic-assisted therapy, ayahuasca ceremony) may attend smaller effect size changes. Increased emotional stability (TIPI Calm Emotionally stable) was only observed between baseline and 2weeks, and thus will not be interpreted. Similarly, because changes in TIPI extraversion did not extend to 4weeks, and its counterpart item, TIPI Reserved, showed no change, this result is not interpreted.

Collectively, our results indicate that psychedelic use may promote personality traits of high relevance to social functioning, namely traits linked to lower reactivity and antagonism in interpersonal communication, relationship stability, and emotion regulation. Results involving decreased neuroticism may be consistent with recent neurocognitive work demonstrating reduced negative affect (and amygdala response) in relation to facial stimuli 1week following psilocybin use, and decreased trait anxiety and increased positive affect 1month following psilocybin use (Barrett et al., 2020).

To evaluate the size of these personality change effects relative to alternative interventions, we additionally compared observed effect size changes in TIPI Anxious and TIPI Critical to meta-analyzed effects of psycho- and pharmacotherapeutic intervention. In their large meta-analysis (k=199; *N*=~20,000), Roberts and colleagues (2017) observed that neuroticism declined and agreeableness increased by 0.57 and 0.15 standard deviations, respectively, in relation to 24week (on average) clinical interventions.^7^ When calculated in an equivalent manner in our sample (N=249), TIPI Anxious exhibited a lower effect size change, namely a reduction of 0.26 standard deviations; whereas TIPI Critical exhibited a larger effect size change, namely a decrease of 0.27 standard deviations. These results may be suggestive that psychedelic use in the general population does not carry the therapeutic effectiveness for reducing neuroticism that can be found in clinical intervention or in other contexts (e.g., psilocybin-assisted therapy, Erritzoe et al., 2018; ayahuasca ceremony, Weiss et al., 2021), but such use may accompany enhanced effectiveness for enhancing agreeableness (or reducing antagonism). Nevertheless, it is important to bear in mind that Roberts and colleagues’ (2017) meta-analysis likely included a small proportion of studies targeting agreeableness specifically, potentially suppressing their observed effect size for agreeableness, and making our effect size estimates appear artifactually favorable.

Although speculative, it may be additionally useful to explore the degree to which the trajectories of change in personality found in this study relate to each other. Covariance of change over time may be diagnostic of common psychedelic-induced processes that underlie multiple traits. To accomplish this goal, we conducted analyses examining correlations of change scores in those traits that exhibited significant change in our study (Table 6). Notably, TIPI Anxious exhibited a medium-sized correlation with TIPI Critical.^8^ Such intraindividual covariation of neuroticism and agreeableness following psychedelic experience may point to lower critical and quarrelsome disposition under psychological conditions of reduced negative emotionality. This relation may also point to common causes involving enhanced emotion regulation ability (e.g., regulation of distress and frustration). Few studies have explored these possibilities in particular. Nevertheless, one previous study showed that men with a history of psychedelic use reported better emotion regulation ability and lower intimate partner violence, and that emotion regulation ability cross-sectionally mediated the relation between psychedelic use and intimate partner violence (Thiessen et al., 2018). Applied to our own work, it is conceivable that reduced neuroticism *via* enhanced emotion regulation accompanies lower stress reactivity and interpersonal aggression, as indexed by our TIPI Critical Quarrelsome item. It will be important for future prospective work to explore how psychedelic use may mechanistically impact BFAS Belligerence (low pole of Politeness) and externalizing behavior, possibly through enhanced emotion regulation, meaning, and fulfillment, and reduced negative emotionality and anger reactivity.

### Is Psychedelic Use Related to Change in Perceptions of Social Connectedness?

Our focus on connectedness to other human beings is part of a larger effort to explore the ways in which psychedelic use may engender enhanced connectedness, broadly construed (Carhart-Harris et al., 2018). Such connectedness is thought to be evident in acute ego dissolution experience by which the coherent self becomes diminutive within awareness and most clearly in acute mystical experiences of oceanic boundlessness and external unity by which one experiences unification with external objects and entities (Barrett et al., 2015). Previous research has shown that psychedelic-induced connectedness also extends to self (Watts et al., 2017), nature (e.g., Forstmann and Sagioglou, 2017; Lyons and Carhart-Harris, 2018) and other human beings (Forstmann et al., 2020). Increases in perceived social connectedness was found using both of our operationalizations: a semantic face-valid measure and a visual measure that asked participants to select the degree of overlap between self and other that best represents their current relationship with other human beings. However, we only interpret changes in SCS Social Connectedness, capturing perceptions of belongingness to the social environment, because changes in Relatedness did not extend to 4weeks post. Nevertheless, it is important to mention that the present study replicated the findings of Forstmann et al. (2020) using a prospective design and the same measure (i.e., Relatedness in the present study). Our results notably differed in demonstrating that the effect of enhanced connectedness with other human beings persisted for up to 2weeks (versus 24h demonstrated in their study).

Our findings may hold important clinical implications. Social disconnectedness is a common feature of internalizing and stress disorders (Karp, 2017; Kintzle et al., 2018), and connectedness is considered a core component of psychological well-being (Lee et al., 2008; Cervinka et al., 2012). Furthermore, social disconnectedness shows concurrent and longitudinal relations with loneliness (Jose and Lim, 2014; Ang, 2016). Accordingly, it will be important for future research to examine perceived social connectedness as a possible psychological mechanism underlying therapeutic psychedelic-induced effects on depression, stress, and loneliness.

### Is Change in Personality Connected to Change in Perceived Social Connectedness?

Adaptive change in perceived social connectedness significantly covaried over time with a number of personality outcomes including neuroticism, extraversion, agreeableness, and conscientiousness. The present approach was not able to evaluate the causal directionality of these relations. Therefore, our results provide only preliminary data with regard to the processes underlying adaptive change. It will be important for future work to explore mechanistically how these change processes interact with each other (e.g., through the use of ecological momentary assessment). As one possibility, enhanced perceived social connectedness may lead to expanded in-group identification, lower distrust, reduced neuroticism, and enhanced extraversion, agreeableness, and conscientiousness. Alternatively, reduced neuroticism and enhanced extraversion and agreeableness may be primary and serve to amplify perceptions of social connectedness as individuals deepen their prosocial involvement in the social environment. Given the rapidity with which perceived social connectedness emerged in previous work (Forstmann et al., 2020), the latter explanation may be less likely as changes in social behavior may not have had significant time to unfold. As a further explanation, interindividual overlap between constructs may be suggestive that observations of covariation over time are merely artifacts of the overlapping component changing in both. We consider this possibility while recognizing that interindividual covariation does not *necessarily* imply intraindividual covariation over time (Molenaar, 2004; Fleeson, 2007). Our results indicated that interindividual overlap with perceived social connectedness in the cases of TIPI Critical and Disorganized was small (|*r|*<0.30), whereas overlap in the cases of TIPI Anxious and Extraverted was more substantial (|*r|*>0.30). This pattern is tentatively suggestive that intraindividual covariation is more likely to be suggestive of distinct constructs with common processes in the former cases, and common constructs in the latter. As a final comment on our results, only SCS Social Connectedness exhibited covarying relations with personality, suggesting that common processes may underlie change in perceived *social belonging* and personality, but not perceived *relatedness to other human beings* (Relatedness) and personality.^9^

### Are There Factors That Predispose or Potentiate Change in Social Functioning-Related Traits and Perceived Social Connectedness?

Four-hundred-thirty-two analyses were conducted to examine the degree to which changes in outcomes were moderated by predisposing and acute factors, with eight significant results emerging (2% of analyses). Although this rate exceeds the Type I error rate expected from setting an alpha level of *p*<0.005 (i.e., 0.5%), the low number of significant results is suggestive that they should be cautiously interpreted as explorative findings warranting future replication. In general, predisposing and acute factors did not emerge within our general population data. Moreover, our hypotheses of moderated change in neuroticism, extraversion, and openness by mystical experience were not supported. Given that our sample was relatively well-powered to interpret a small interaction effect in most cases, it is important to consider why our null results differ so starkly from other studies that observed greater evidence of moderation (e.g., MacLean et al., 2011; Erritzoe et al., 2018; Weiss et al., 2021). One possibility is that use in the general population—lacking the control and introspective attentional buffers of psychedelic-assisted and ceremonial contexts—includes greater variability in other factors not measured here (e.g., relationship dynamics among participating users) that increase unsystematic variance in change trajectories. Another possibility is that the online volunteer format of survey response impacted the fidelity with which participants rated their experiences, though no evidence exists to this effect. A third possibility is that previous work has overstated the influence of predisposing and acute factors on psychedelic-induced personality change.

## Limitations

There are a number of limitations that qualify the interpretation of our findings. First, perhaps the larger source of validity concern regards possible differential drop out by participants who did not experience a positive effect of psychedelics. Only 22% of early participants completed all timepoints, and 34% completed baseline and 2weeks post. As such, the present sample may underreport participant trajectories that would more heavily favor null hypotheses. Future research that reduces such attrition is vital for testing the validity of preliminary findings found in our sample. Second, comparisons of complete and incomplete respondents were suggestive of differences in conscientiousness. As such, attrition-related sample bias could have impacted results pertaining to conscientiousness; in addition, attrition-related sample bias could conceivably extend beyond measured outcomes, and limit generalizability. Third, the naturalistic approach of the present study precluded the use of a control group, raising the potential for significant methodological issues including the influence of placebo, expectancy, and demand effects, especially among those who are advocates for expanded psychedelic use. Third, informant-data was not collected in this study to corroborate results from self-report data. Although self-report data represents a valid source of information, previous work has shown that informant-report data tends to support only a subset of significant personality change results found in self-report data (Weiss et al., 2021). The presence of strong placebo and expectancy effects in this work make informant-data an important source of information. Fourth, our sample was comprised of volunteers, meaning that sample bias may have played a role in excluding individuals for whom enrollment would have required compensation; this property may partially limit generalizability to the broader population. Fifth, our use of TIPI items accompanies low inter-item reliability of personality outcomes, and behooves the importance of future replication, especially in the case of results where only one of two intra-domain TIPI items showed a significant effect (e.g., extraversion). Sixth, our survey study was not able to precisely measure the type and dosage of the substances participants took. Given evidence of dose-dependent effects of psychedelics (e.g., Holze et al., 2021b), this introduces imprecision into our estimates of longer-term change, making our results significantly less generalizable to particular use cases.

## Conclusion

The present study represented a well-powered examination of how psychedelic use in a naturalistic setting may influence social functioning and connectedness using the framework of FFM personality. Use of psychedelics was associated with substantive decreases in neuroticism and increases in agreeableness, both of which are relevant to social functioning *via* links to relationship satisfaction and prosociality. Specific adaptive effects on personality involved changes in anxiety, emotional lability, quarrelsomeness, and enthusiasm. These data add to the robustness of findings involving a psychedelic-induced antidepressant effect and shows evidence of this effect extending to the general population of psychedelic users. Our data also provide preliminary evidence for an intriguing downward effect on a particular aspect of FFM agreeableness, namely BFAS Belligerence (or Politeness at the opposite pole), that involves a conflict-prone style of interpersonal relating. Use of psychedelics was also associated with substantive increases in feelings of belonging to one’s social environment. Predisposing and acute factors were generally not associated with change trajectories, though baseline standing on neuroticism, extraversion, perspective taking, and perceived social connectedness showed qualified evidence of amplifying adaptive effects on these traits. Our findings are broadly suggestive of the therapeutic potential of psychedelics for addressing Antagonistic Externalizing and loneliness.

## Data Availability Statement

The datasets presented in this article are not readily available because, but inquiries for responsible use of this data can be made to DE. Requests to access the datasets should be directed to d.erritzoe@imperial.ac.uk.

## Ethics Statement

The studies involving human participants were reviewed and approved by Imperial College Research Ethics Committee; Joint Research Compliance Office. The patients/participants provided their written informed consent to participate in this study.

## Author Contributions

BW wrote this paper with feedback and editing from DE, RC-H, VN, and LP. All authors read and approved the final manuscript.

## Funding

The authors received no financial support for the research, authorship, and/or publication of this article. RC-H is supported by the Alex Mosley Charitable Trust.

## Conflict of Interest

The authors declare that the research was conducted in the absence of any commercial or financial relationships that could be construed as a potential conflict of interest.

## Publisher’s Note

All claims expressed in this article are solely those of the authors and do not necessarily represent those of their affiliated organizations, or those of the publisher, the editors and the reviewers. Any product that may be evaluated in this article, or claim that may be made by its manufacturer, is not guaranteed or endorsed by the publisher.

## Footnotes

^1^Hubner, S., Haijen, E., Kaelen, M., Carhart-Harris, R., and Kettner, H. (under review). Turn on, tune in, and drop out: identifying predictors of attrition in observational psychedelic research. Journal of Medical Internet Research.

^2^ www.psychedelicsurvey.com

^3^ www.reddit.com/r/LSD

^4^ www.reddit.com/r/Psychonaut7

^5^ www.surveygizmo.eu

^6^As a note, results from the present study are not directly commensurable with Mason et al.’ (2019) finding at 7 days as cognitive and affective empathy was measured using a laboratory test (Multifaceted Empathy Test) that elicits empathic ability rather than relying on participants’ self-appraisal of empathic ability.

^7^Strictly pharmacological interventions produced a change in neuroticism of 0.48 standard deviations.

^8^It bears noting, TIPI Calm did not exhibit a similar pattern.

^9^However, it bears noting that power analyses demonstrated we were limited in detecting true covariation between the latter set of constructs; our sample size provided 80% power to detect a correlation between change scores exceeding 0.28.
