# Supplementary material for: Examining Psychedelic-Induced Changes in Social Functioning and Connectedness in a Naturalistic Online Sample Using the Five-Factor Model of Personality
Source: Front Psychol. 2021 Nov 25;12:749788. doi: 10.3389/fpsyg.2021.749788 (PMC8655335; doi:10.3389/fpsyg.2021.749788)
Supplement: Supplementary file 1 [file Data_Sheet_1.docx]

**Supplementary Materials**

**Supplementary Figures**


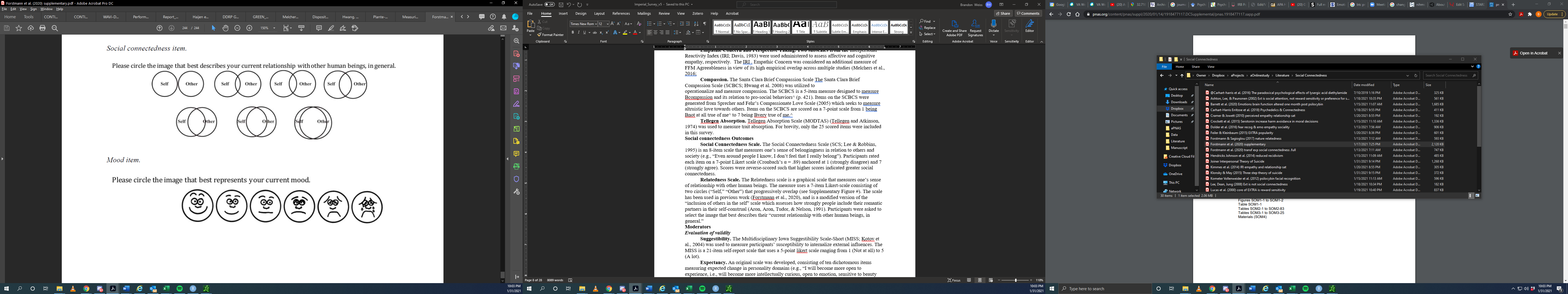


Supplementary Figure 1. Display of single-item Relatedness scale


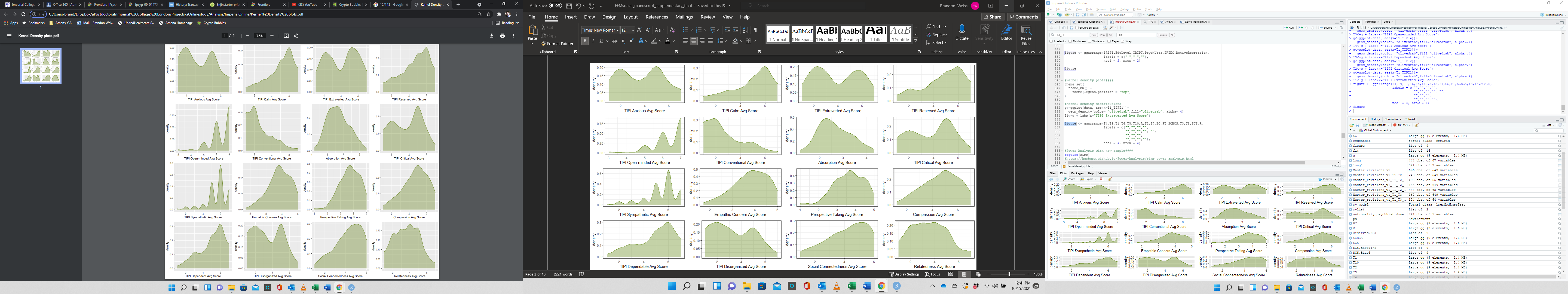


Supplementary Figure 2. Kernel density plots of baseline personality and social connectedness outcome scores

Supplementary Tables

Supplementary Table 1.

Zero-order correlations between baseline personality and social connectedness outcomes

| Outcome | 1 | 2 | 3 | 4 | 5 | 6 | 7 | 8 | 9 | 10 | 11 | 12 | 13 | 14 | 15 |
| --- | --- | --- | --- | --- | --- | --- | --- | --- | --- | --- | --- | --- | --- | --- | --- |
| 1. TIPI Anxious |  |  |  |  |  |  |  |  |  |  |  |  |  |  |  |
| 2. TIPI Calm | -.59** |  |  |  |  |  |  |  |  |  |  |  |  |  |  |
| 3. TIPI Extraverted | -.31** | .13' |  |  |  |  |  |  |  |  |  |  |  |  |  |
| 4. TIPI Reserved | .28** | -.03 | -.64** |  |  |  |  |  |  |  |  |  |  |  |  |
| 5. TIPI Open-minded | -.19** | .14' | .30** | -.09 |  |  |  |  |  |  |  |  |  |  |  |
| 6. TIPI Conventional | .15' | -.13' | -.19** | .11 | -.24** |  |  |  |  |  |  |  |  |  |  |
| 7. Tellegen Absorption | .02 | .11 | .17* | -.03 | .23** | -.20** |  |  |  |  |  |  |  |  |  |
| 8. TIPI Critical | .24** | -.21** | .01 | .03 | .00 | .08 | .02 |  |  |  |  |  |  |  |  |
| 9. TIPI Sympathetic | -.06 | .11 | .18** | -.12 | .13' | -.15' | .20** | -.19** |  |  |  |  |  |  |  |
| 10. IRI Empathic Concern | -.05 | .08 | .20** | -.14' | .17* | -.10 | .30** | -.14' | .42** |  |  |  |  |  |  |
| 11. IRI Perspective Taking | -.18** | .26** | .21** | -.12 | .19** | -.16* | .27** | -.24** | .33** | .50** |  |  |  |  |  |
| 12. SCBCS Compassion | -.12 | .09 | .22** | -.20** | .14' | -.03 | .29** | -.14' | .42** | .76** | .48** |  |  |  |  |
| 13. TIPI Dependable | -.24** | .32** | .10 | -.07 | .23** | -.13' | .08 | -.16* | .11 | .10 | .09 | .08 |  |  |  |
| 14. TIPI Disorganized | .22** | -.17* | -.04 | .09 | -.06 | .03 | .08 | .21** | .14' | .01 | .08 | .04 | -.39** |  |  |
| 15. SCS Social Connected | -.45** | .36** | .41** | -.40** | .16' | -.17* | -.06 | -.24** | .26** | .23** | .31** | .23** | .08 | -.11 |  |
| 16. Relatedness | -.20** | .16' | .24** | -.27** | .08 | .00 | .16* | -.09 | .23** | .37** | .26** | .32** | -.02 | .08 | .48** |
| *Note*. TIPI=Ten-Item Personality Inventory; Sample used is N = 249; SCBCS=Santa Clara Brief Compassion Scale; SCS=Social Connectedness Scale; ‘*p*=.05, **p*<.01, ***p*<.005. | | | | | | | | | | | | | | | |

Supplementary Table 2.

Full Significant Results of Linear Mixed Models

| Predictor / Moderator | Parameter | B | Std Error | t-value | *p*-value |
| --- | --- | --- | --- | --- | --- |
| **Neuroticism** | | | | | |
| Anxious, easily upset (TIPI 4) | | | | | |
| Main effect of Time | Intercept | 3.69** | .15 | 24.96 | .000 |
| T1.T2.T3 n = 148 | Time 2 weeks | -.49** | .11 | -4.42 | .000 |
|  | Time 4 weeks | -.55** | .11 | -4.96 | .000 |
| Main effect of Time | Intercept | 3.51** | .12 | 30.02 | .000 |
| T1.T2 n = 249 | Time 2 weeks | -.40** | .09 | -4.45 | .000 |
| Main effect of Time | Intercept | 3.72** | .14 | 26.84 | .000 |
| T1.T3 n = 162 | Time 4 weeks | -.56** | .11 | -4.99 | .000 |
| Baseline TIPI 4 | Intercept | 3.69** | .08 | 44.81 | .000 |
|  | Time 2 weeks | -.49** | .10 | -4.80 | .000 |
|  | Time 4 weeks | -.55** | .10 | -5.39 | .000 |
|  | Baseline TIPI 4 | 1.72** | .08 | 22.72 | .000 |
|  | Moderator X Time 2 weeks | -.49** | .10 | -4.73 | .000 |
|  | Moderator X Time 4 weeks | -.75** | .10 | -7.29 | .000 |
| Calm, emotionally stable (TIPI 9) | | | | | |
| Main effect of Time | Intercept | 5.00** | .12 | 40.43 | .000 |
| T1.T2.T3 n = 148 | Time 2 weeks | .24 | .10 | 2.48 | .014 |
|  | Time 4 weeks | .25* | .10 | 2.62 | .009 |
| Main effect of Time | Intercept | 5.08** | .09 | 54.36 | .000 |
| T1.T2 n = 249 | Time 2 weeks | .22* | .08 | 2.78 | .006 |
| Main effect of Time | Intercept | 5.01** | .12 | 41.23 | .000 |
| T1.T3 n = 162 | Time 4 weeks | .22 | .10 | 2.24 | .026 |
| Knowledge Bias | Intercept | 5.00** | .12 | 40.74 | .000 |
|  | Time 2 weeks | .24 | .09 | 2.52 | .012 |
|  | Time 4 weeks | .25* | .09 | 2.67 | .008 |
|  | Knowledge Bias | .37** | .12 | 2.99 | .003 |
|  | Moderator X Time 2 weeks | -.28** | .09 | -2.98 | .003 |
|  | Moderator X Time 4 weeks | -.29** | .09 | -3.10 | .002 |
| Experienced Bias | Intercept | 5.00** | .12 | 41.73 | .000 |
|  | Time 2 weeks | .24 | .09 | 2.52 | .012 |
|  | Time 4 weeks | .25* | .09 | 2.67 | .008 |
|  | Experienced Bias | .56** | .12 | 4.63 | .000 |
|  | Moderator X Time 2 weeks | -.23 | .09 | -2.46 | .014 |
|  | Moderator X Time 4 weeks | -.34** | .09 | -3.57 | .000 |
| Baseline TIPI 9 | Intercept | 5.00** | .07 | 72.89 | .000 |
|  | Time 2 weeks | .24* | .08 | 2.79 | .006 |
|  | Time 4 weeks | .25** | .08 | 2.95 | .003 |
|  | Baseline TIPI 9 | 1.65** | .07 | 24.03 | .000 |
|  | Moderator X Time 2 weeks | -.64** | .08 | -7.54 | .000 |
|  | Moderator X Time 4 weeks | -.67** | .08 | -7.92 | .000 |
| **Extraversion** | | | | | |
| Extraverted, enthusiastic (TIPI 1) | | | | | |
| Main effect of Time | Intercept | 3.90** | .15 | 26.84 | .000 |
| T1.T2.T3 n = 148 | Time 2 weeks | .25 | .11 | 2.35 | .020 |
|  | Time 4 weeks | .18 | .11 | 1.71 | .088 |
| Main effect of Time | Intercept | 4.08** | .11 | 35.72 | .000 |
| T1.T2 n = 249 | Time 2 weeks | .23* | .09 | 2.64 | .009 |
| Main effect of Time | Intercept | 3.91** | .14 | 27.36 | .000 |
| T1.T3 n = 162 | Time 4 weeks | .19 | .10 | 1.84 | .068 |
| Reserved, quiet (TIPI 6) | | | | | |
| Main effect of Time | Intercept | 4.82** | .13 | 36.10 | .000 |
| T1.T2.T3 n = 148 | Time 2 weeks | -.12 | .10 | -1.22 | .224 |
|  | Time 4 weeks | -.09 | .10 | -.88 | .380 |
| Main effect of Time | Intercept | 4.71** | .11 | 44.57 | .000 |
| T1.T2 n = 249 | Time 2 weeks | -.11 | .08 | -1.46 | .146 |
| Main effect of Time | Intercept | 4.75** | .13 | 35.90 | .000 |
| T1.T3 n = 162 | Time 4 weeks | -.07 | .10 | -.69 | .494 |
| EBI | Intercept | 4.8** | .14 | 34.70 | .000 |
|  | Time 2 weeks | -0.10 | .09 | -1.09 | .278 |
|  | Time 4 weeks | -0.05 | .09 | -.54 | .587 |
|  | EBI 6 | .17 | .14 | 1.20 | .230 |
|  | Moderator X Time 2 weeks | -.18 | .09 | -1.95 | .052 |
|  | Moderator X Time 4 weeks | -.34** | .09 | -3.66 | .000 |
| **Openness** | | | | | |
| Open to new experiences, complex (TIPI 5) | | | | | |
| Main effect of Time | Intercept | 6.36** | .08 | 82.21 | .000 |
| T1.T2.T3 n = 148 | Time 2 weeks | -.06 | .08 | -.73 | .468 |
|  | Time 4 weeks | -.18 | .08 | -2.18 | .030 |
| Main effect of Time | Intercept | 6.36** | .05 | 118.68 | .000 |
| T1.T2 n = 249 | Time 2 weeks | -.07 | .06 | -1.17 | .245 |
| Main effect of Time | Intercept | 6.34** | .07 | 84.62 | .000 |
| T1.T3 n = 162 | Time 4 weeks | -.15 | .09 | -1.80 | .073 |
| Conventional, uncreative (TIPI 10) | | | | | |
| Main effect of Time | Intercept | 2.50** | .12 | 21.58 | .000 |
| T1.T2.T3 n = 148 | Time 2 weeks | -.06 | .10 | -.60 | .551 |
|  | Time 4 weeks | .03 | .10 | .33 | .741 |
| Main effect of Time | Intercept | 2.47** | .09 | 27.43 | .000 |
| T1.T2 n = 249 | Time 2 weeks | .01 | .08 | .14 | .886 |
| Main effect of Time | Intercept | 2.51** | .12 | 21.63 | .000 |
| T1.T3 n = 162 | Time 4 weeks | .04 | .10 | .42 | .674 |
| Tellegen Absorption | | | | | |
| Main effect of Time | Intercept | 2.65** | .06 | 46.16 | .000 |
| T1.T2.T3 n = 148 | Time 2 weeks | -.01 | .03 | -.41 | .680 |
|  | Time 4 weeks | -.06 | .03 | -2.46 | .014 |
| Main effect of Time | Intercept | 2.66** | .04 | 60.59 | .000 |
| T1.T2 n = 249 | Time 2 weeks | .01 | .02 | .28 | .778 |
| Main effect of Time | Intercept | 2.65** | .05 | 49.41 | .000 |
| T1.T3 n = 162 | Time 4 weeks | .06 | .03 | -2.36 | .020 |
| **Agreeableness** | | | | | |
| Critical, quarrelsome (TIPI 2) | | | | | |
| Main effect of Time | Intercept | 3.74** | .14 | 26.15 | .000 |
| T1.T2.T3 n = 148 | Time 2 weeks | -.47** | .11 | -4.12 | .000 |
|  | Time 4 weeks | -.56** | .11 | -4.88 | .000 |
| Main effect of Time | Intercept | 3.75** | .11 | 34.33 | .000 |
| T1.T2 n = 249 | Time 2 weeks | .43** | .09 | -4.78 | .000 |
| Main effect of Time | Intercept | 3.70** | .14 | 27.08 | .000 |
| T1.T3 n = 162 | Time 4 weeks | -.44** | .11 | -3.92 | .000 |
| Sympathetic, warm (TIPI 7) | | | | | |
| Main effect of Time | Intercept | 5.47** | .10 | 55.21 | .000 |
| T1.T2.T3 n = 148 | Time 2 weeks | .22* | .08 | 2.61 | .010 |
|  | Time 4 weeks | .08 | .08 | .00 | 1.000 |
| Main effect of Time | Intercept | 5.58** | .07 | 75.52 | .000 |
| T1.T2 n = 249 | Time 2 weeks | .13 | .06 | 2.06 | .041 |
| Main effect of Time | Intercept | 5.48** | .09 | 58.32 | .000 |
| T1.T3 n = 162 | Time 4 weeks | -.01 | .08 | -.16 | .872 |
| IRI Empathic Concern | | | | | |
| Main effect of Time | Intercept | 3.79** | .06 | 62.53 | .000 |
| T1.T2.T3 n = 148 | Time 2 weeks | -.02 | .03 | -.62 | .535 |
|  | Time 4 weeks | -.02 | .03 | -.62 | .535 |
| Main effect of Time | Intercept | 3.83** | .05 | 84.96 | .000 |
| T1.T2 n = 249 | Time 2 weeks | -.00 | .03 | -.02 | .986 |
| Main effect of Time | Intercept | 3.79** | .09 | 58.32 | .000 |
| T1.T3 n = 162 | Time 4 weeks | -.03** | .06 | 64.81 | .000 |
| IRI Perspective Taking | | | | | |
| Main effect of Time | Intercept | 3.63** | .06 | 57.20 | .000 |
| T1.T2.T3 n = 148 | Time 2 weeks | .04 | .03 | 1.15 | .253 |
|  | Time 4 weeks | .05 | .03 | 1.60 | .111 |
| Main effect of Time | Intercept | 3.70** | .05 | 75.90 | .000 |
| T1.T2 n = 249 | Time 2 weeks | -.00 | .03 | .07 | .944 |
| Main effect of Time | Intercept | 3.64** | .06 | 61.16 | .000 |
| T1.T3 n = 162 | Time 4 weeks | -.05 | .04 | 1.37 | .173 |
| Lifetime Psychedelic uses | Intercept | 3.63** | .06 | 57.86 | .000 |
|  | Time 2 weeks | .04 | .03 | 1.17 | .245 |
|  | Time 4 weeks | .05 | .03 | 1.63 | .105 |
|  | Lifetime Psychedelic uses | .19** | .06 | 3.03 | .003 |
|  | Moderator X Time 2 weeks | -.11** | .03 | -3.17 | .002 |
|  | Moderator X Time 4 weeks | -.09** | .03 | -2.83 | .005 |
| Baseline IRI PT | Intercept | 3.63** | .03 | 134.361 | .000 |
|  | Time 2 weeks | .04 | .03 | 1.22 | .223 |
|  | Time 4 weeks | .05 | .03 | 1.71 | .089 |
|  | Baseline IRI PT | .80** | .03 | 29.64 | .000 |
|  | Moderator X Time 2 weeks | -.12** | .03 | -3.78 | .000 |
|  | Moderator X Time 4 weeks | -.21** | .03 | -6.48 | .000 |
| Santa Clara Brief Compassion Scale | | | | | |
| Main effect of Time | Intercept | 4.66** | .11 | 41.71 | .000 |
| T1.T2.T3 n = 148 | Time 2 weeks | .00 | .06 | .02 | .982 |
|  | Time 4 weeks | -.08 | .06 | -1.29 | .198 |
| Main effect of Time | Intercept | 4.73** | .09 | 55.12 | .000 |
| T1.T2 n = 249 | Time 2 weeks | -.02 | .05 | .31 | .760 |
| Main effect of Time | Intercept | 4.71** | .11 | 43.14 | .000 |
| T1.T3 n = 162 | Time 4 weeks | -.10 | .06 | -1.59 | .113 |
| **Conscientiousness** | | | | | |
| Dependable, self-disciplined (TIPI 3) | | | | | |
| Main effect of Time | Intercept | 5.03** | .12 | 40.42 | .000 |
| T1.T2.T3 n = 148 | Time 2 weeks | .09 | .11 | .87 | .385 |
|  | Time 4 weeks | .11 | .11 | 1.06 | .291 |
| Main effect of Time | Intercept | 5.08** | .09 | 54.14 | .000 |
| T1.T2 n = 249 | Time 2 weeks | .16 | .09 | 1.87 | .063 |
| Main effect of Time | Intercept | 5.07** | .12 | 43.67 | .000 |
| T1.T3 n = 162 | Time 4 weeks | .10 | .10 | .95 | .342 |
| Disorganized, careless (TIPI 8) | | | | | |
| Main effect of Time | Intercept | 3.25** | .14 | 23.68 | .000 |
| T1.T2.T3 n = 148 | Time 2 weeks | .02 | .11 | .19 | .848 |
|  | Time 4 weeks | -.10 | .11 | -.96 | .338 |
| Main effect of Time | Intercept | 3.36** | .11 | 31.41 | .000 |
| T1.T2 n = 249 | Time 2 weeks | .17 | .09 | -1.96 | .052 |
| Main effect of Time | Intercept | 3.28** | .13 | 24.93 | .000 |
| T1.T3 n = 162 | Time 4 weeks | .14 | .10 | -1.38 | .169 |
| **Social Connectedness** | | | | | |
| Social-Connectedness Scale | | | | | |
| Main effect of Time | Intercept | 4.17** | .11 | 38.80 | .000 |
| T1.T2.T3 n = 148 | Time 2 weeks | .17 | .09 | 1.95 | .052 |
|  | Time 4 weeks | .21 | .09 | 2.35 | .020 |
| Main effect of Time | Intercept | 4.26** | .08 | 53.34 | .000 |
| T1.T2 n = 249 | Time 2 weeks | .19* | .07 | 2.69 | .008 |
| Main effect of Time | Intercept | 4.20** | .10 | 41.53 | .000 |
| T1.T3 n = 162 | Time 4 weeks | .18* | .07 | 2.70 | .008 |
| Knowledge Bias | Intercept | 4.17** | .12 | 40.74 | .000 |
|  | Time 2 weeks | .17 | .09 | 2.52 | .012 |
|  | Time 4 weeks | .21* | .09 | 2.67 | .008 |
|  | Knowledge Bias | .26** | .12 | 2.99 | .003 |
|  | Moderator X Time 2 weeks | -.23** | .09 | -2.98 | .003 |
|  | Moderator X Time 4 weeks | -.04** | .09 | -3.10 | .002 |
| Relatedness to Others Scale | | | | | |
| Main effect of Time | Intercept | 3.48** | .12 | 28.52 | .000 |
| T1.T2.T3 n = 148 | Time 2 weeks | .27* | .10 | 2.77 | .006 |
|  | Time 4 weeks | .22 | .10 | 2.29 | .023 |
| Main effect of Time | Intercept | 3.61** | .09 | 38.17 | .000 |
| T1.T2 n = 249 | Time 2 weeks | .30 | .09 | 3.45 | .001 |
| Main effect of Time | Intercept | 3.51** | .12 | 29.48 | .000 |
| T1.T3 n = 162 | Time 4 weeks | .23 | .10 | 2.20 | .029 |
| Baseline Relatedness | Intercept | 3.48** | .07 | 47.19 | .000 |
|  | Time 2 weeks | .27** | .09 | 3.05 | .002 |
|  | Time 4 weeks | .22 | .09 | 2.52 | .012 |
|  | Baseline Relatedness | 1.54** | .08 | 20.32 | .000 |
|  | Moderator X Time 2 weeks | -.60** | .09 | -6.72 | .000 |
|  | Moderator X Time 4 weeks | -.63** | .09 | -7.10 | .000 |
| Note. B=unstandardized effect size; **p*<.05; ***p*<.01. | | | | | |
